# Erythema Multiforme and COVID-19: What Do We Know?

**DOI:** 10.3390/medicina57080828

**Published:** 2021-08-16

**Authors:** Luigi Bennardo, Steven Paul Nisticò, Stefano Dastoli, Eugenio Provenzano, Maddalena Napolitano, Martina Silvestri, Maria Passante, Cataldo Patruno

**Affiliations:** 1 Department of Health Sciences, Magna Graecia University of Catanzaro, 88100 Catanzaro, Italy; steven.nistico@gmail.com (S.P.N.); stefanodastoli@gmail.com (S.D.); martinaeg@hotmail.it (M.S.); maria.passante@studenti.unicz.it (M.P.); cataldopatruno@libero.it (C.P.); 2 Unit of Dermatology, Mariano Santo Hospital, 87100 Cosenza, Italy; eprovenzano0@gmail.com; 3 Department of Health Sciences V. Tiberio, University of Molise, 86100 Campobasso, Italy; maddy.napolitano@gmail.com

**Keywords:** COVID-19, SARS-CoV-2, erythema multiforme, hydroxychloroquine

## Abstract

*Background*: Erythema multiforme (EM) is an acute cutaneous eruption often associated with infections and more rarely with drugs. This review aimed to evaluate the association between erythema multiforme and coronavirus disease 2019 (COVID-19). *Methods*: A systematic search of PubMed/MEDLINE, Scimago Scopus, and ISI/Web of Science was performed. Original articles, case series, or case reports were evaluated and selected. *Results*: Fourteen articles were selected, describing a total of 70 patients. EM is a cutaneous eruption rarely occurring in COVID-19 and is, in most cases, associated with a hypersensitivity reaction to the virus. In these cases, EM seems to affect patients younger than 30 years or older than 55 years. Infrequently, some drugs used in the management of COVID-19 may induce EM, especially hydroxychloroquine. The three groups of patients seem to have different clinical characteristics and courses. *Conclusions*: From these data, it is possible to preliminarily propose that EM or EM-like eruptions linked to COVID-19 might be divided into three types: the virus-related juvenile type (affecting patients <30-year-old), the virus-related older type (affecting patients >55 years), and the drug-induced type. The occurrence of a skin rash does not seem to be related to the severity and clinical course of COVID-19.

## 1. Introduction

Erythema multiforme (EM) is a skin disease characterized by the appearance, usually on the extremities but often also occurring in other skin areas, of reddish, annular macules, which later become papules, sometimes coalescing to plaques. Often, the earliest EM lesions have a target appearance, with a central portion that can appear as a dusky area, surrounded by a dark red inflammatory zone and another lighter ring on the extreme periphery [1]. EM lesions usually evolve, leading to geographic, polycyclic, and annular configurations. In severe cases, the oral, ocular, or genital mucosa can also be involved. The clinical pattern of EM may be atypical, i.e., an unusual distribution or a lack of the target appearance, and is therefore sometimes called an EM-like eruption. Furthermore, the histological aspect can also be variable, consisting of inflammatory perivascular and interface infiltration, hyperkeratosis, granulation tissue, mucinosis, and acanthosis [2]. Therefore, EM’s clinical and histological variability can likely be considered in the context of a different spectrum of the same pathology.

EM is mainly associated with infectious diseases and rarely with drug exposure [3]. The primary pathogens associated with EM are herpesviruses and Mycoplasma pneumoniae, and, less frequently, other viruses, such as parvovirus B19 or adenoviruses. Recently, severe acute respiratory syndrome coronavirus 2 (SARS-CoV-2) infection (coronavirus disease 2019; COVID-19) has been linked to EM or EM-like eruptions [4,5]. This review aimed to analyze the current state of the art regarding this co-occurrence. A possible classification of COVID-19-related EM is also suggested. 

## 2. Materials and Methods

In order to collect articles, a search in the electronic databases PubMed, Embase, and Cochrane Skin databases was performed up to 31 December 2020, using the term “erythema multiforme” or “erythema multiforme-like” in combination with “COVID-19” or “SARS-CoV-2”. We screened 88 contributions. Only original articles, case series, or case reports were included. All the screened articles’ full texts were carefully examined and evaluated independently by all the authors. Only the documents for which there was complete agreement were selected. At the end of this evaluation procedure, 14/88 (15.9%) articles (8 case reports, 2 case series, and 4 original studies) were included in the review.

## 3. Results

Seventy (31 males, 44.29%; aged 1–95 years) patients with EM or EM-like eruption were reported in the selected articles. In most cases (67/70, 95.71%), the skin disease was strongly associated with a viral infection. These patients were aged <30 (44/73, 60.27%) or >55 (23/7, 32.86%) years old. In only 3/70 (4.29%) patients, the skin rash was associated with drugs, although not definitely in all cases.

### 3.1. Virus-Induced EM and EM-like Eruption in Patients Aged <30 Years Old

Usually, in young patients, the rash was described as mild and was mainly located on the palms and soles [6]. A study analyzing 132 patients infected by SARS-CoV-2 and with various types of acute palmoplantar skin lesions found that 37 (28.03%) of them had EM-like lesions [7]. The age ranged from 1 to 29 years, with a low mean age (12 years). The clinical aspect was different from conventional EM. Indeed, the individual lesions were small and rarely had a targetoid appearance. Furthermore, skin sites other than palmoplantar (elbows, knees, and ears) were involved in only 2/37 (5.4%) of them [7]. Torrello et al. found EM-like eruptions with histologic findings of mild perivascular dermatitis in 4/22 (18.2%) children affected by COVID-19-related chilblains [6]. Generally, EM or EM-like eruptions in younger patients were not associated with a more severe course of COVID-19, even in cases of diffuse skin involvement [8]. However, EM has also been described in a 13-old boy affected by “pediatric multi-system inflammatory syndrome temporally related to COVID-19”, characterized by fever for several days, inflammatory multi-system involvement, and elevated inflammation biomarkers. Interestingly, EM was one of the first signs of the syndrome in this patient and disappeared concomitantly to the fever [9].

### 3.2. Virus-Induced EM and EM-Like Eruption in Patients Aged >55 Years Old

EM and EM-like eruptions associated with SARS-CoV-2 infection have also been described in older patients. A Spanish study of 375 patients described EM-like eruptions among the other cutaneous symptoms in COVID-19 patients. There were 17/375 (4.5%) subjects, the mean age was 61.5 years, and 88.2% were female. The eruptions were mainly erythematous papules and target lesions. The rash was generalized (70.6%), symmetrical (47.1%), confluent (41.2%), and/or palmoplantar (11.8%). The eruption started on the trunk (70.6%) or upper limbs (23.5%). The mean duration of the skin condition was 9.7 ± 4.9 days. In about two-thirds of the cases, the rash was symptomatic, with itching (72.7%) and a burning sensation (27.3%) as the main symptoms [10].

Gargiulo et al. reported the case of a 72-year-old woman who developed a severe form of EM before the onset of respiratory symptoms and positivity to a reverse transcription polymerase chain reaction test for SARS-CoV-2. Unlike other reported cases, both skin and systemic symptoms did not respond well to corticosteroids and other therapy for COVID-19, and the patient died [11].

A case series described four women aged between 58 and 77 years. Three of them manifested EM after clinical resolution of COVID-19, with typical skin findings and mild mucosal involvement. All the cases healed after 2 to 3 weeks of systemic corticosteroid therapy [4]. On the other hand, one case of EM with exclusive mucosal ocular and oral involvement was described in a 57-year-old male; a course of oral steroids led the patient to remission of the mucosal symptoms [12].

### 3.3. Drug-Induced EM and EM-Like Eruption

EM or EM-like eruption has been only rarely associated with drug intake [3]. Currently, there are no cases of these skin eruptions undoubtedly associated with the treatments for COVID-19. Even in patients with such a viral infection and exposed to various drugs, differentiating the cause of EM or EM-like eruptions is difficult. For example, Reguero et al. reported a 95-year-old female patient who manifested EM lesions 2 days after hospital discharge following SARS-CoV-2 infection and a 10-day cycle of hydroxychloroquine; in this case, the authors did not relate the EM to drugs [13]. However, in some cases, EM and EM-like eruptions have been associated with the current treatment for COVID-19. Any age can be involved. Janah et al. reported two cases of EM associated with SARS-CoV-2 infection [14]. The first patient was a 17-year-old boy in which EM appeared on the extremities and was likely associated with the systemic disease. On the other hand, they also described a 29-year-old male patient with targetoid lesions in the palms during treatment with hydroxychloroquine. Both cases spontaneously resolved in a couple of weeks. The latter case was resolved without suspending the drug, so it might also be considered an EM secondary to viral infection and not related to hydroxychloroquine assumption. 

Dermibas et al. described the case of a 37-year-old woman who reported the appearance of severe EM with mucosal involvement 5 days after starting treatment of COVID-19 pneumonia with hydroxychloroquine and oseltamivir. The patient suspended all therapies and was treated with high doses of corticosteroids, leading to the healing of both pneumonia and EM. The authors linked the eruption to the systemic infection; notwithstanding, the temporal involvement may also suggest that hydroxychloroquine or oseltamivir could cause EM [15].

Robustelli et al. reported the case of a 70-year-old woman treated with hydroxychloroquine and lopinavir/ritonavir, reporting an eruption characterized by targetoid pustular lesions finally diagnosed as atypical acute generalized exanthematous pustulosis (AGEP) with an EM-like pattern, an occurrence already reported during hydroxychloroquine therapy [16].

## 4. Discussion

Both EM and EM-like eruptions have been reported as being mainly associated with SARS-CoV-2 infection or, much more rarely, induced by the disease’s treatment. These skin eruptions’ pathophysiological mechanism could be a hypersensitivity reaction mediated by lymphocytes targeting SARS-CoV-2 antigens in the skin, similar to what was reported for EM associated with other infections. In particular, CD8+ T lymphocytes induce the apoptosis of scattered keratinocytes and lead to satellite cell necrosis [1]. Furthermore, the prevalence of the acral involvement could suggest Type III and/or IV hypersensitivity targeting the small vessels of the skin responsible for endothelial activation, dermal, and perivascular lymphoid infiltration. The consequence is an interface dermatitis and exocytosis of lymphocytes. A dermal infiltrate typically shows lymphohistiocytic infiltration surrounding the superficial and mid-dermal vessels [1,2]. Histological observations seem to corroborate this hypothesis [17]. Virus-induced EM has been primarily reported in young (<30 years old) and older (>55 years old) patients.

In younger patients, the skin rash is usually located at the extremities, especially on the palms and soles. Often, the clinical appearance is atypical [7]. In some patients, it is associated with COVID-19-related chilblains [6]. In one case, EM was one of the first signs of “pediatric multi-system inflammatory syndrome temporally related to COVID-19”. However, in most cases, EM and EM-like eruptions were not related to a severe evolution of COVID-19. Since most juvenile cases of COVID-19 are mild, EM or EM-like eruption does not appear to be a marker of disease severity.

EM and EM-like eruptions in older patients seem to be more frequent in women, more severe, and to have more variable clinical aspects. Surprisingly, in these patients, the eruption may sometimes appear after the resolution of the respiratory symptoms. The rash is mainly located on the trunk and then spreads to other body areas, often symmetrically. Most of the cases described had an excellent prognosis both of EM and COVID-19 [10]. In one case, the occurrence of cutaneous before systemic symptoms and the inadequate response to systemic corticosteroid treatment was associated with a worse prognosis of COVID-19 [11]. Although isolated, this case could lead one to believe that the lack of response to therapy for severe EM during COVID-19 could be considered a negative prognostic sign. However, based on the currently available data, it does not seem that there is a direct relationship between cutaneous and systemic disease severity. Besides, EM is known to not always respond to corticosteroid treatment [1].

Drug-induced EM in COVID-19 patients has been related to hydroxychloroquine and has been reported to appear 3 to 10 days after starting the treatment. Drug discontinuation and systemic corticosteroid led to resolving the symptoms [18]. A case also reported the possible link between EM appearance and azithromycin [19]. The association between the mRNA-1273 (Moderna) COVID-19 vaccine and bullous drug eruption resembling EM has been described [20]. Hydroxychloroquine may also induce AGEP, characterized by a generalized, pustular, and figurate rash, thus clinically resembling EM [21].

## 5. Conclusions

EM is a well-known dermatologic condition mostly associated with infections. Notwithstanding, EM and EM-like eruptions linked with COVID-19 seem to have some peculiar characteristics. Indeed, the currently available data from the literature suggest that they may be divided into three types (Table 1). (1) The virus-related juvenile type (sometimes associated with chilblains) usually involves patients aged <30 years, usually involving the extremities, with palmoplantar involvement. (2) The virus-related older type (>55 years of age) involves mainly females; the onset of skin eruption in most cases follows pulmonary involvement and spreads from the trunk to the rest of the body. (3) The drug-induced type, mainly due to hydroxychloroquine and usually appearing after some days from starting the treatment; the suspension of the drug and an oral course of steroids usually lead to resolution. However, studies on a larger population, evaluating histologic and pathogenetic aspects, are needed to identify better the various subtypes and the clinical significance of EM and EM-like eruptions associated with COVID 19.

## Figures and Tables

**Table 1:** Clinical characteristics of COVID-19-associated erythema multiforme (EM).

Clinical Subtype	Virus-Related Juvenile Type	Drug-Induced Type	Virus-Related Older Type
Age	<30 years of age	>30 years of age	>55 years of age
Clinical characteristics	No systemic involvement Acral distribution	Treatment with hydroxychloroquine	Spread from the trunk to the rest of the body.
Histology	Mild superficial perivascular dermatitis, without necrosis and eosinophils	Infiltration of CD8+ lymphocytes into the epidermis, resulting in keratinocyte necrosis and subepidermal blistering	Normal stratum corneum, mild to moderate spongiosis in the epidermis. Dilated vessels filled with neutrophils, extravasation of red blood cells, and lymphocytic perivascular and interstitial infiltrate in the dermis

## Data Availability

The study did not report any new data.
